# Macromolecular crowding has opposite effects on two critical sub-steps of transcription initiation

**DOI:** 10.1002/1873-3468.14851

**Published:** 2024-03-13

**Authors:** Pratip Mukherjee, Abhishek Mazumder

**Affiliations:** 1 Structural Biology and Bioinformatics Division, CSIR-Indian Institute of Chemical Biology, Kolkata, India; 2 Academy of Scientific and Innovative Research (AcSIR), CSIR-Human Resource Development Centre, Ghaziabad, India

**Keywords:** macromolecular crowding, photoisomerisation-induced fluorescence enhancement, promoter escape, RNA polymerase, transcription initiation

## Abstract

Transcription initiation, the first step in gene expression, has been studied extensively in dilute buffer, a condition which fails to consider the crowded environment in live cells. Recent reports indicate the kinetics of promoter escape is altered in crowded conditions for a consensus bacterial promoter. Here, we use a real-time fluorescence enhancement assay to study the kinetics of unwound bubble formation and promoter escape for three separate promoters. We find that the effect of crowding on transcription initiation is complex, with lower rates of unwound bubble formation, higher rates of promoter escape, and large variations depending on promoter identity. Based on our results, we suggest that altered conditions of crowding inside a live cell can trigger global changes.

For many decades, experiments performed in aqueous buffer solutions have been instrumental in understanding the mechanism of a biochemical reaction. However, in a live cell, the environment is different due to conditions of crowding generated by the presence of large concentrations of proteins, nucleic acids, carbohydrates etc., (~ 80–400 mg·mL^−1^; [1–3]), which occupy up to 40% of cellular volume [3]. Such crowded environments lead to an increase in viscosity which results in slower macromolecular motions and kinetics [4]. Additionally, it produces an “excluded volume effect” or in other words a decrease in space available for diffusion of a molecule [5–10], and a concomitant increase in the effective concentration of molecules. The thermodynamic consequence of the “excluded volume effect” is a significant decrease in entropy which affects the conformational energy landscape navigated by biological macromolecules as they tend to minimise the “excluded volume” effect by changes to their structure (compaction) or association with other partners [11–17]. Consistent with this, *in vitro* studies mimicking conditions of macromolecular crowding with crowders like polyethylene glycol (PEG)/Dextran/Ficoll etc., show significant impact on the conformational equilibria, interactions, and activity of biological macromolecules [14–17]. Importantly, the extent of crowding in a cell can change depending on environmental conditions, like osmotic upshifts, nutrient starvation etc., and can also potentially vary between individual cells. Along with enhanced viscosity and the excluded volume effect, the crowded molecular environment inside a cell can also potentially result in other weak or strong molecular interactions influencing biological activity.

Transcription, the first step in gene expression has been studied in detail using biochemical and biophysical methods *in vitro* by carrying out reactions using reconstituted transcription complexes in dilute aqueous buffer [18]. In the last decade, researchers working with cell-free expression systems using a T7 RNA polymerase (RNAP) reported enhanced transcription rates in a crowded environment and attributed it to an enhancement of the RNAP-promoter association rate, which they assumed to be rate determining [19,20]. More recently, a study monitoring rate of RNA production at the single molecule level reported altered rates of promoter escape in solutions containing large crowders like PEG-8000 for transcription from a consensus bacterial promoter [21]. Additionally, several reports in the last few years have emerged identifying formation of droplets *via* liquid–liquid phase separation at transcription sites in both eukaryotic and bacterial cells, indicating altered microenvironments and changes to crowding conditions formed around sites of transcription may be a common phenomenon across all kingdoms of life [22–25]. It is therefore imperative to understand how crowding impacts the process of transcription.

In this work, we investigate how crowding affects the kinetics of promoter unwinding and promoter escape, two crucial sub-steps in transcription initiation for three characteristically different promoter fragments – a consensus bacterial promoter (lacCONS), a phage λ promoter (pR), and a ribosomal RNA promoter (rrnBP1). We use a photoisomerisation-induced fluorescence enhancement (PIFE) assay [26–28] for monitoring promoter unwinding and promoter escape reactions in real-time in presence or absence of a large crowder – PEG-8000, which has a hydrodynamic radius of ~ 2.45 nm [6], and mimics macromolecular crowding conditions encountered *in vivo* [2,29]. Our results show that these two sub-steps in transcription initiation are affected in opposite ways with crowded conditions resulting in reduced rates of promoter unwinding and enhanced rates of promoter escape. Interestingly, our results indicate the extent of deceleration of unwinding rates and enhancement of escape rates were dramatically different depending on the promoter.

## Materials and methods

### *E. coli* RNAP core enzyme

Hexa-histidine tagged RNAP was prepared as follows: 10 mL LB containing 100 μg·mL^−1^ ampicillin was inoculated with single colonies of *E. coli* strain BL21(DE3) transformed with pVS10 plasmid and incubated overnight at 37 °C with shaking at 180 r.p.m. 10 mL overnight culture was used to inoculate 1 L LB broth containing 100 μg·mL^−1^ ampicillin and incubated at 37 °C with shaking at 180 r.p.m. until OD_600_ reached 0.7. IPTG was then added to a final concentration of 1 mm, and culture was further incubated at 37 °C with shaking at 180 r.p.m. for 4 h. Cells were harvested by centrifugation at 4000 ***g*** for 30 min, resuspended in 70 mL Buffer A [50 mm Tris–HCl (pH 7.9), 200 mm NaCl, 5% glycerol] containing 1 EDTA free protease inhibitor tablet (Roche Diagnostics, Mannheim, Germany), incubated on ice for 15 min, lysozyme was added to a final concentration of 1 mg·mL^−1^ and further incubated on ice for 30 min. Cells were disrupted by ultrasonication for 1 min (20 cycles) with 3 min cooling intermissions. Cell debris was removed by centrifugation (20000 ***g***, 30 min at 4 °C), supernatant was collected, and to the supernatant, 10% (v/v) solution of PEI (pH 7.9) was added to a final concentration of 0.35% at 4 °C over 10 min. Solution was stirred for 10 min, followed centrifugation for 15 min at 12000 ***g*** and supernatant was discarded. Pellet was resuspended in 50 mL Buffer B (10 mm Tris–HCl, pH 7.9, 0.5 m NaCl and 5% glycerol), stirred for 15 min at 4 degrees, followed by centrifugation at 12000 ***g*** for 15 min. The supernatant was discarded, and the pellet was resuspended in 50 mL Buffer C (10 mm Tris–HCl, pH 7.9, 1 M NaCl, and 5% glycerol), stirred for 30 min at 4 °C, followed by centrifugation for 15 min at 12000 ***g*** and supernatant was collected. Ammonium sulphate (17.5 g) was slowly added to 50 mL supernatant collected in the previous step at 4 °C, solution was stirred gently for 45 min at 4 °C, followed by centrifugation at 17000 ***g*** for 40 min. Supernatant was discarded and the pellet was dissolved in 30 mL buffer D (10 mm Tris–HCl, pH 7.9, 200 mm NaCl, and 5% glycerol) and loaded onto a 5 mL Ni-NTA-agarose column pre-equilibrated in buffer D and incubated for 2 h. Column was washed with 50 mL buffer D containing 10 mm imidazole and eluted with 25 mL buffer D containing 200 mM imidazole. The sample was further purified by anion-exchange chromatography on a HiTrap Q HP (Cytiva, GE Healthcare Biosciences, Uppsala, Sweden; 160 mL linear gradient of 300–500 mm NaCl in 10 mm Tris–HCl, pH 7.9, 0.1 mm EDTA and 5% glycerol; flow rate = 2 mL·min^−1^). Fractions containing RNAP core enzyme were pooled and concentrated using 30 kDa MWCO Amicon Ultra-15 centrifugal ultrafilters. RNAP core enzyme was exchanged into storage buffer (25 mm Tris–HCl, pH 7.9, 100 mm NaCl, 0.1 mm EDTA, and 5% glycerol) using Amicon Ultra-15 centrifugal ultrafilters.

### σ^70^

σ^70^ was prepared as in [30].

### *E. coli* RNAP holoenzyme

RNAP core (3.4 μm) was mixed with σ^70^ (10.2 μm) and incubated at 30 °C for 30 min to prepare the RNAP holoenzyme.

### Nucleic acids

Oligodeoxyribonucleotides were purchased from Biomers, Ulm, Germany. Non-template strand oligodeoxyribonucleotide (2 mm), and template strand oligodeoxyribonucleotide (2.2 mm) in 50 μL 10 mm Tris–HCl, pH 7.9 and 0.2 m NaCl were heated 5 min at 95 °C, cooled to 25 °C in 1 °C steps with 0.5 min per step using a thermal cycler (MiniAmp plus; Thermo Fisher, Waltham, MA, USA), and stored at −20 °C. Promoter sequences for [lacCONS-Cy3], [rrnBP1-Cy3] and [pR-Cy3] are described in Table S1.

### Photoisomerisation-induced fluorescence enhancement (PIFE) assay

For all PIFE experiments, excitation wavelength was 550 nm (slit width was 5 nm), and emission was set at 570 nm (slit width was 5 nm) with integration time of 1 s. Data was collected for 30 s for endpoint experiments and 1000 s for real-time experiments. All experiments were repeated three times.

### Endpoint experiments

Endpoint PIFE assays were performed by measuring fluorescence intensity values of the (a) promoter-Cy3 DNA alone in 1× transcription buffer (TB; 20 mm Tris–HCl pH 7.9, 10 mm MgCl_2_, 100 mm NaCl, 5 mm b-Mercaptoethanol, 5% glycerol, and 100 mg·mL^−1^ BSA) or 1× crowding buffer (CB; 20 mm Tris–HCl pH 7.9, 10 mm MgCl_2_, 100 mm NaCl, 5 mm b-Mercaptoethanol, 5% glycerol, 10% PEG-8000 and 100 mg·mL^−1^ BSA); (b) RNAP-promoter open complexes (RPo) formed after incubation of RNAP with promoter-Cy3 DNA for 40 min in 1× TB or 1× CB; and (c) after incubation of RPo obtained from (b) with NTPs and heparin leading to promoter escape in 1× TB or 1× CB.

### Real-time experiments

For the promoter unwinding assay, 30 μL of 20 nM [lacCONS-Cy3] or [rrnBP1-Cy3] or [pR-Cy3] in 1× TB or 1× CB was taken in fluorescence cuvette and 30 μL of 200 nM RNAP holoenzyme in 1× TB (or 1× CB) was added by manual mixing and Cy3 fluorescence intensity was observed using PTI fluorimeter. The time course of intensity change was fit to the following biexponential equation:

(1) $$y=A_{1}+A_{2}\times \left[1-e_{\text{fast}\;}^{-k}\times x\right]+A_{3}\times \left[1-e_{\text{slow}\;}^{-k}\times x\right]$$

where, *y* = (*F*/*F*_0−1_), *F* is the fluorescence intensity and *F*_0_ the fluorescence intensity at *x* = 0; and *x* = time in seconds. As per the results of endpoint experiments, we should observe a ~ 2-fold increase in intensity resulting in change of *y* from 0 to 1. However, due to manual mixing (dead time of ~ 3–5 s) the signal corresponding to *x* = 0 represents a timepoint which is ~ 3–5 s after the experiment starts. Therefore, the extent of the observed enhancement in the real-time experiments is less than expected.

For the promoter escape assay, the RNAP-promoter open complex (RPo) was prepared by incubating 60 μL of 20 nm [lacCONS-Cy3] or [rrnBP1-Cy3] or [pR-Cy3], and 200 nm RNAP holoenzyme in 1× TB (or 1× CB) at 37 °C for 20 min. The RPo solution was transferred to a fluorescence cuvette, 60 μL of 400 μm each ATP, GTP, CTP, UTP and 100 μg·mL^−1^ heparin in 1× TB (or 1× CB) was added by manual mixing, and fluorescence intensity was monitored on a PTI fluorimeter (time resolution: 1 s). Excitation was 550 nm, and emission was set at 570 nm. The time course of intensity change was fit to the following biexponential equation:

(2) $$y=A_{1}+A_{2}\times \left[e_{\text{fast}}^{-k}\times x\right]+A_{3}\times \left[e_{\text{slow}}^{-k}\times x\right]$$

where, *y* = (*F*/*F*_0_), *F* is the fluorescence intensity and *F*_0_ the fluorescence intensity at *x* = 0; and *x* = time in seconds. Control experiments were performed by mixing 60 μL of RPo complexes with 60 μL of 100 μg·mL^−1^ heparin in 1× TB. All experiments were repeated at least three times and the mean, and the standard error of mean were calculated. All Data analysis were performed in ORIGIN 2018 (Origin Labs, Northampton, MA, USA).

### Estimation of viscosities and viscosity-adjusted rate constants

First, we determined the viscosity of 1× TB or 1× CB relative to water using fluorescence correlation spectroscopy (FCS). We used 20 nm Alexa 647 maleimide and took FCS measurements in water, 1× TB or 1× CB. The FCS measurements were carried out using an ISS Alba FFS/FLIM confocal system (Champaign, IL, USA), coupled to a Nikon Ti2U microscope equipped with the Nikon CFI PlanApo 60×/1.2NA water immersion objective. Excitation was performed with a 640-nm picosecond pulsed diode laser and emission were collected using a SPAD (Single Photon Avalanche Detector) with a 650-nm long-pass filter. The FCS correlation curves were fit to the 3D Gaussian 1-component diffusion model, to obtain the diffusion time (τ_D_) of Alexa 647 maleimide in water (τ_A647-water_), 1× TB (τ_A647-TB_), and 1× CB (τ_A647-CB_). Since τ_D_ is directly proportional to the viscosity (η) at the same temperature and detection volume, we used the following relations to estimate the bulk viscosity of buffer solutions:

(3) $$\eta_{\text{buffer}\;}=\eta_{\text{water}}\times \left(\tau_{\text{A647}-\text{buffer}}/\tau_{\text{A647}-\text{water}}\right)$$

where, η_buffer_ is the viscosity of buffer solution used (1× TB or 1× CB), η_water_ is viscosity of water, τ_A647-buffer_ is the diffusion time of Alexa 647 maleimide in the buffer solution used (1× TB or 1× CB), and τ_A647-water_ is the diffusion time of Alexa 647 maleimide in water. We estimated the bulk viscosities in 1× TB (η_TB_) and 1× CB (η_CB_) to be 1.78-fold and 7.8-fold higher compared to η_water_. Further, PEG-8000 has a hydrodynamic radius ~ 2.45 nm smaller than the overall size of RNAP-promoter complexes used in this study and we therefore assume micro-viscosities experienced by such complexes are same as the bulk viscosity of the medium [31].

Kramers’ kinetic theory implies that the kinetics is inversely related to the viscosity. This has been demonstrated for processes like protein folding [32]. We assume the same relation holds true for the kinetic constants measured in this study and estimate viscosity-adjusted rate constants using the following general relation:

(4) $$k_{\text{adjusted}\;}=k_{\text{observed}}\times \left(\eta_{\text{buffer}}/\eta_{\text{water}\;}\right)$$

where, *k*_adjusted_ is the viscosity-adjusted rate constant and *k*_observed_ is the overall rate constant obtained directly from the experiments.

## Results

### PIFE for investigating the sub-steps of transcription initiation in presence of crowders

**Fig. 1:**
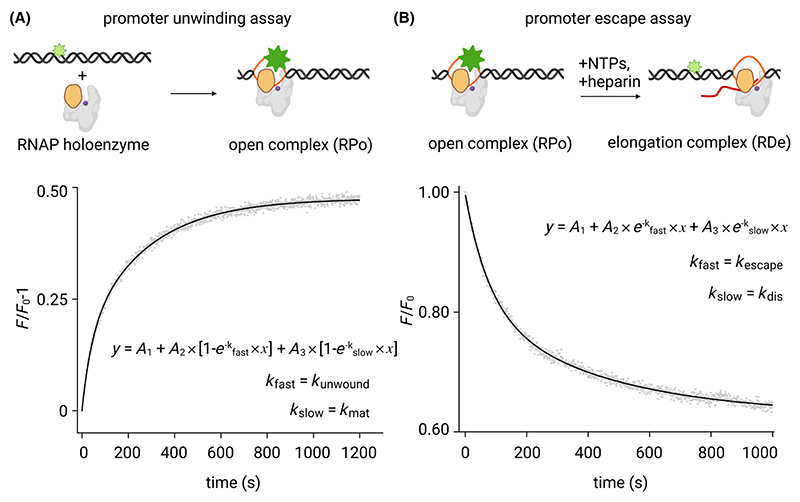
PIFE assay for monitoring promoter unwinding and promoter escape. (A) (Top) Design of experiment monitoring promoter unwinding in real time. Grey, RNAP; orange, RNAP clamp; purple dot, RNAP active-centre; black, ds-DNA; orange, ss-DNA; light green, Cy3 on ds-DNA; dark green, Cy3 on ss-DNA. (Bottom) Representative time course of relative fluorescence intensity change (grey dots) from Cy3 attached to the promoter bubble of a consensus bacterial promoter, lacCONS obtained after manual mixing of RNAP holoenzyme and [lacCONS-Cy3] in 1× TB. Time resolution: 1 s; excitation: 550 nm; emission: 570 nm; Solid line represents nonlinear regression fit to a biexponential function (inset). Experiments were repeated three times. (B) (Top) Design of experiment monitoring promoter escape in real time. Colours as in 1A, and red, RNA. (Bottom) Representative time course of relative fluorescence intensity change (grey dots) from Cy3 attached to the promoter bubble of a consensus bacterial promoter, lacCONS obtained after manual mixing of RNAP-promoter open complex (RPo) with 200 μm each ATP, GTP, CTP, UTP and 50 μg·mL^−1^ heparin in 1× TB. Time resolution: 1 s; excitation: 550 nm; emission: 570 nm; Solid line represents nonlinear regression fit to a biexponential function (inset). Experiments were repeated three times.

To investigate the role of macromolecular crowding on the sub-steps of transcription initiation, we employed a PIFE assay to monitor promoter unwinding and promoter escape using the fluorescence probe, Cy3, which when attached to a double-stranded promoter bubble segment, exhibits an ~ 2-fold increase in fluorescence intensity (PIFE) as the RNAP binds and unwinds the promoter bubble to form the RPo ([26–28]; Fig. 1A). We attached Cy3 to the promoter bubble segment for three prototypical promoter fragments: (a) a consensus bacterial promoter [lacCONS], (b) a naturally occurring promoter with a 17-bp spacer and an AT-rich downstream promoter sequence [λ-pR], and (c) a naturally occurring promoter with a 16-bp spacer and a GC-rich downstream promoter segment [rrnBP1] to generate three labelled promoter constructs, [lacCONS-Cy3], [pR-Cy3], and [rrnBP1-Cy3] (Table S1). In case of [lacCONS-Cy3] and [rrnBP1-Cy3] the fluorescent probe was placed at downstream end of the promoter bubble, while for [pR-Cy3] the probe was placed at the upstream end. All three constructs ensure we observe the formation of a fully unwound bubble since in case of lacCONS and rrnBP1 the upstream bubble segment unwinds before the downstream segment (Mazumder and Kapanidis, unpublished), while for pR upstream and downstream unwinding occur together in a single step ([33]; Mazumder and Kapanidis, unpublished).

First, we performed endpoint PIFE assays and measured fluorescence intensity values of the promoter constructs before incubation with RNAP, after ~ 40 min incubation with RNAP to form RPo, and after incubation of RPo with NTPs leading to promoter escape in TB or CB. Observed intensity values for the three promoters and RNAP-promoter complexes were similar in TB and CB indicating conditions of crowding by PEG-8000 did not affect the brightness of the fluorophore (Fig. S1A). Results of these experiments show enhanced intensity values after formation of RPo and a decrease in intensity values after incubation of RPo with NTPs for the labelled promoter constructs in all three cases (Fig. S1). RPo formation resulted in enhancement by ~ 2.3-fold for lacCONS, ~ 1.9-fold for pR and ~ 2.1-fold for rrnBP1 (Fig. S1), and promoter escape resulted in a significant decrease with measured fluorescence intensity values of ~ 1.25-fold for lacCONS, ~ 1.24-fold for pR, and ~ 1.03-fold for rrnBP1 compared to the baseline values observed for promoter only (Fig. S1). Notably, in all cases the intensity values do not return to the same baseline values, suggesting not all molecules forming the RPo proceed to promoter escape – an observation in agreement with previous results indicating some complexes are stuck in the abortive initiation phase of initial transcription (Fig. S1; [26]). Taken together, these experiments show that our ensemble PIFE assay could be used to faithfully monitor promoter unwinding from the fluorescence enhancement observed after RNAP binding and bubble formation, and promoter escape from the fluorescence decrease after initial transcription and bubble collapse.

To assess the role of macromolecular crowding on PIFE and RNAP activity, identical experiments were performed in a CB containing PEG-8000 (10%) in TB. Results of endpoint PIFE experiments in CB revealed results similar to those obtained in TB with intensity values of ~ 2.1-fold, ~ 1.8-fold and ~ 2.2-fold after RPo formation and intensity values of ~ 1.1-fold, ~ 1.2-fold, and ~ 1.2-fold after promoter escape for lacCONS, pR and rnnBP1promoters, indicating a crowded molecular environment did not affect the extent of intensity changes, did not alter RNAP activity and did not significantly change the fraction of complexes stuck in abortive initiation for any of the three promoters studied (Fig. S1).

### The rate of unwound bubble formation is decreased in a crowded environment

Next, we monitored promoter unwinding in real-time by monitoring intensity values after manual mixing of a Cy3-promoter fragment with RNAP holoenzyme in TB (Fig. 1A). The results show an intensity vs time trajectory with rapid increase in fluorescence intensity which reaches a steady intensity value in a few mins (Fig. 1A). The observed intensity change reflects an increase in the RPo molecules produced and the rate of this increase is influenced by the individual rate constants for all sub-steps including binding of RNAP to the promoter, reversible unwinding of the promoter, and subsequent conformational changes involving the RNAP clamp [27], and/or other downstream mobile elements [34,35]. Our experiments have a typical dead time of ~ 3–5 s due to manual mixing of the components and therefore we miss the very fast initial promoter-RNAP binding step which occur at time-scales of <1 s [36] – and is typically not a rate-determining step in transcription initiation. Subsequent steps following binding including promoter recognition, promoter unwinding, and other conformational changes resulting in a mature open complex are captured in our assay.

The data obtained from PIFE assays for all three promoters fit best to a biexponential distribution resulting in estimation of two rate constants: a fast component, *k*_fast_ and a slow component, *k*_slow_ (Fig. 1A, bottom panel; Figs S2 and S3). The increase in fluorescence intensity results from a step of reversible unwinding of the promoter bubble and subsequent environmental changes experienced by Cy3 as a mature RPo forms. Typical values of the fast component, *k*_fast_, were in the range of ~ 0.02–0.07 s^−1^ and were close to previously estimated values for promoter unwinding in single molecule assays for the same three promoters (~ 0.09–0.14 s^−1^ for lacCONS, pR and rrnBP1; [27], Mazumder and Kapanidis, unpublished). The observed intensity increase in our assay reflects the rate of unwound bubble accumulation and as such depends on both rates of unwinding and rate of rewinding. Therefore, it is expected that the rate of accumulation of unwound bubbles would be slightly lower than the actual rates for unwinding estimated from the single molecule assay. It is therefore reasonable to assign the fast component, *k*_fast_ to the rate of reversible unwound bubble formation (*k*_fast_ = *k*_unwound_). On the other hand, typical values of *k*_slow_ were in the range of ~ 0.003–0.004 s^−1^, an order of magnitude slower than the expected values of promoter unwinding and therefore we assign this to changes associated with the formation of a mature RPo after the unwound bubble if formed (*k*_slow_ = *k*_mat_).

**Fig. 2:**
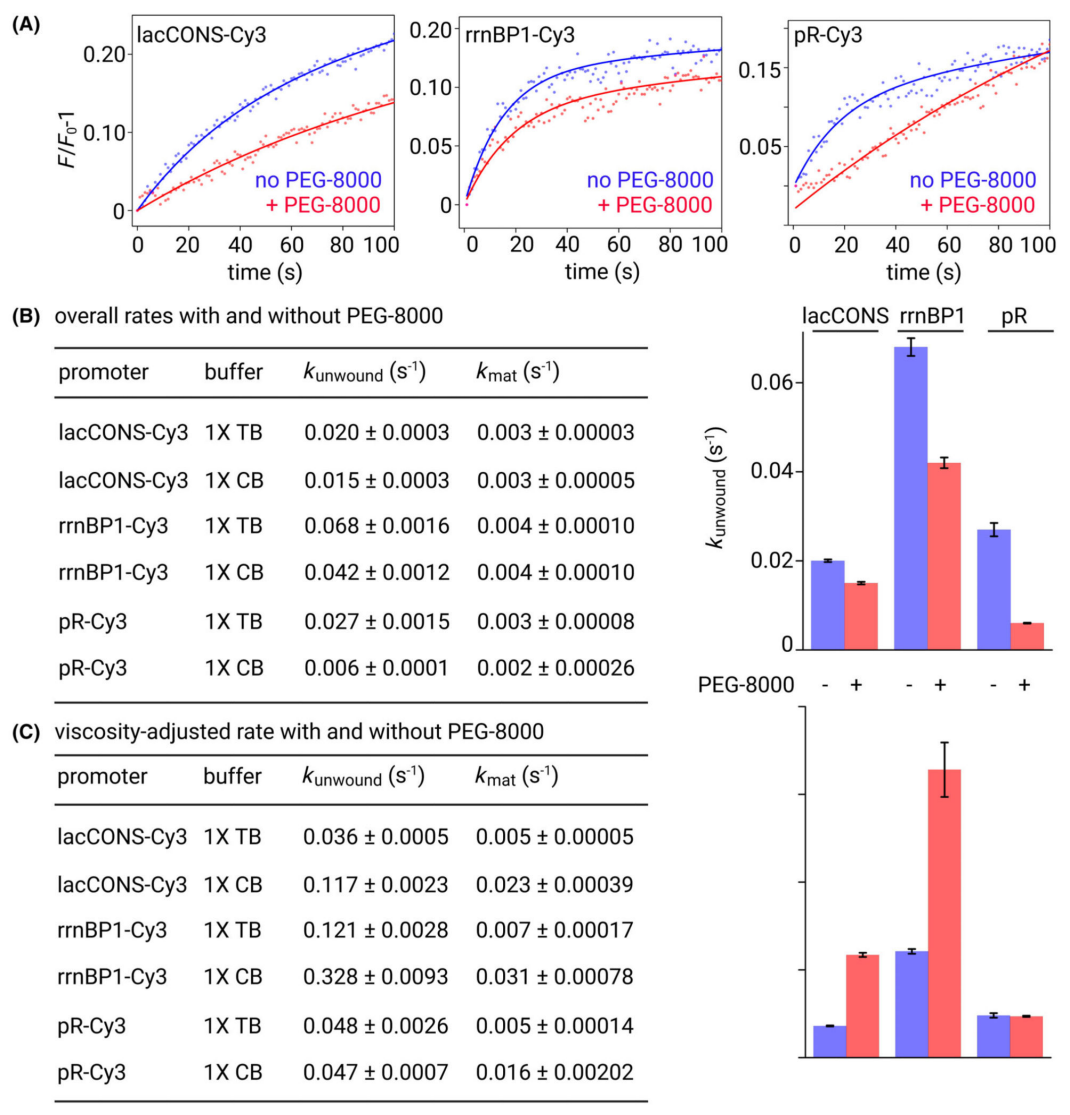
Effect of crowding on rate of unwound bubble formation for three different promoters. (A) Representative time course (up to first 100 s) of relative fluorescence intensity changes from Cy3 attached to the promoter bubble of a consensus bacterial promoter, lacCONS (left), rrnBP1 (middle), pR (right) obtained after manual mixing of RNAP holoenzyme and promoter fragments in 1× TB (blue dots) or 1× CB containing 10% PEG-8000 as the crowder (CB; red dots). Time resolution: 1 s; excitation: 550 nm; emission: 570 nm; Solid lines represent nonlinear regression fit to a biexponential function for experiments performed in 1× TB (blue lines) and 1× CB (red lines). Full time courses are shown in Fig. S3. Experiments were repeated three times. (B) Rates of unwound bubble formation (*k*_unwound_) for lacCONS, rrnBP1 and pR promoters in absence (blue bars) and presence (red bars) of 10% PEG-8000. Table showing the estimated mean rates (with errors) of unwound bubble formation (*k*_unwound_) and RPo maturation (*k*_mat_) in 1× TB and 1× CB for the three promoters. Experiments were repeated three times, error bars are showing standard deviations. (C) Viscosity-adjusted rates of unwound bubble formation (*k*_unwound_) for lacCONS, rrnBP1 and pR promoters in absence (blue bars) and presence (red bars) of 10% PEG-8000. Table showing the estimated mean viscosity-adjusted rates (with errors) of unwound bubble formation (*k*_unwound_) and RPo maturation (*k*_mat_) in 1× TB and 1× CB for the three promoters. Error bars are showing standard deviations.

Intensity vs time trajectories for all three promoters were analysed to estimate the rates of unwound bubble formation and RPo maturation in absence and presence of 10% PEG-8000 (Fig. 2A). The rate of unwound bubble formation in TB varied ~ 4 fold between the three promoters with *k*_unwound_ values of ~ 0.020 s^−1^, ~ 0.027 s^−1^, and ~ 0.068 s^−1^ for lacCONS, pR, and rrnBP1 promoters respectively (Fig. 2A,B). In contrast the rate of RPo maturation was similar with *k*_mat_ values of ~ 0.003 s^−1^, 0.003 s^−1^, and 0.004 s^−1^ for lacCONS, pR, and rrnBP1 (Fig. 2B). The same experiment carried out under conditions of macromolecular crowding in CB resulted in slower rates of unwound bubble formation with *k*_unwound_ values of ~ 0.015 s^−1^, ~ 0.006 s^−1^, and ~ 0.042 s^−1^ for lacCONS, pR, and rrnBP1, respectively. Notably, the extent to which unwinding rates were affected in CB varied widely depending on the promoter sequence from an ~ 1.3-fold change for lacCONS to an ~ 4.5-fold change for pR (Fig. 2B). The rate of RPo maturation, however, remained similar in CB (Fig. 2B), indicating the later stages of mature RPo formation remain unaffected in the presence of crowders.

It is important to note that conditions of crowding lead to a concomitant increase in viscosity, and this results in an overall slowdown of processes like protein folding which are inversely correlated with viscosity [4,32]. Assuming a similar inverse correlation of viscosity with rates of unwound bubble formation, we calculated viscosity-adjusted rates to gain further insight into the effect of excluded volume effect on unwound bubble formation (see Materials and Methods). The viscosity-adjusted rates of unwound bubble formation in 1× TB were ~ 0.03 s^−1^, ~ 0.12 s^−1^, and ~ 0.05 s^−1^, and in 1× CB were ~ 0.12 s^−1^, ~ 0.33 s^−1^, and ~ 0.05 s^−1^, for lacCONS, rrnBP1 and pR promoters, indicating that the excluded volume effect produced by the crowded environment resulted in moderately enhanced rates for lacCONS (~ 3.3-fold) and rrnBP1 (~ 2.7-fold), but produced no significant effect on pR (Fig. 2C).

### The rate of promoter escape is enhanced in a crowded environment

To monitor promoter escape kinetics in real time, we performed real-time promoter escape experiments (see Materials and Methods). For these experiments, we observed a rapid decrease in intensity values followed by a slow and gradual decrease (Fig. 1B, Fig. S4). The intensity vs time trajectories were fit to a biexponential function to extract two rates: a fast component, *k*_fast_ and a slow component, *k*_slow_ (Figs 1B, Figs S2 and S4). Control experiments with only 50 μg·mL^−1^ heparin revealed a slow and gradual decrease in intensity like the slow component described for the previous experiment (Fig. S5), suggesting this phenomenon results from dissociation of RNAP-promoter complexes in presence of the polyanionic heparin which competes with promoter DNA preventing any reassociation of RNAP and promoter DNA. Therefore, we assign the fast component, *k*_fast_ as the rate of promoter escape (*k*_escape_) and the slow component, *k*_slow_ as the rate of RNAP-promoter dissociation (*k*_dis_) – which essentially reflect the stability of RPo.

**Fig. 3:**
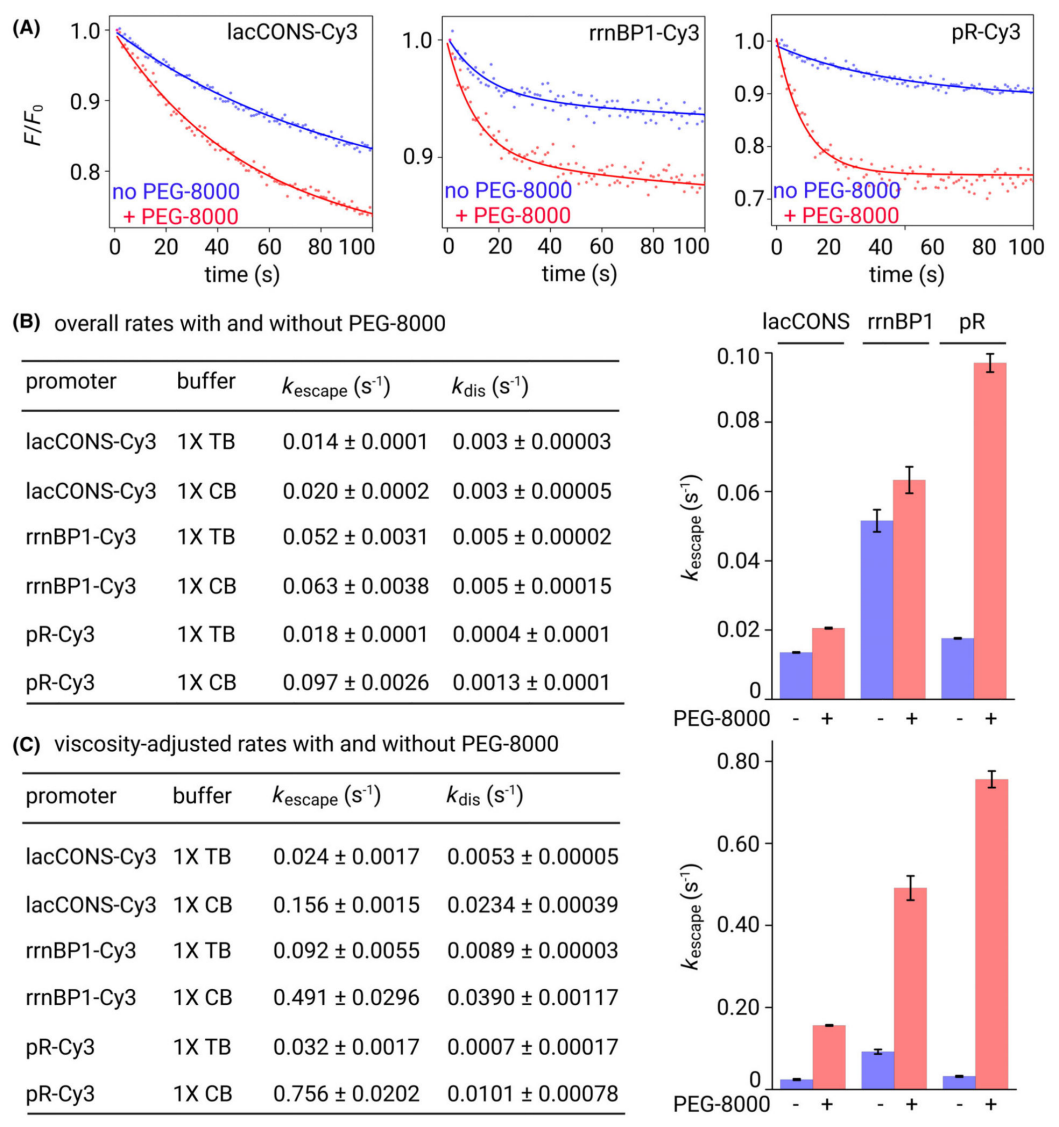
Effect of crowding on rate of promoter escape for three different promoters. (A) Representative time course (up to first 100 s) of relative fluorescence intensity changes from Cy3 attached to the promoter bubble of a consensus bacterial promoter, lacCONS (left), rrnBP1 (middle), pR (right) obtained after manual mixing of RNAP-promoter open complex (RPo) with 200 μm each ATP, GTP, CTP, UTP and 50 μg·mL^−1^ heparin in 1× TB (blue dots) or 1× CB containing 10% PEG-8000 as the crowder (CB; red dots). Time resolution: 1 s; excitation: 550 nm; emission: 570 nm; Solid lines represent nonlinear regression fit to a biexponential function for experiments performed in 1× TB (blue lines) and 1× CB (red lines). Experiments were repeated three times. (B) Rates of promoter escape (*k*_escape_) for lacCONS, rrnBP1 and pR promoters in absence (blue bars) and presence (red bars) of 10% PEG-8000. Table showing the estimated mean rates (with errors) of promoter escape (*k*_escape_) and RNAP-promoter complex dissociation (*k*_dis_) in 1× TB and 1× CB for the three promoters. Experiments were repeated three times, error bars are showing standard deviations. (C) Viscosity-adjusted rates of promoter escape (*k*_escape_) for lacCONS, rrnBP1 and pR promoters in absence (blue bars) and presence (red bars) of 10% PEG-8000. Table showing the estimated mean viscosity-adjusted rates (with errors) of promoter escape (*k*_escape_) and RNAP-promoter complex dissociation (*k*_dis_) in 1× TB and 1× CB for the three promoters. Error bars are showing standard deviations.

Intensity vs time trajectories for all three promoters were analysed to estimate the rates of promoter escape and RNAP-promoter dissociation in absence and presence of 10% PEG-8000 (Fig. 3A). The estimated values of *k*_dis_ for the three promoters were ~ 0.003 s^−1^, ~ 0.0004 s^−1^ and 0.005 s^−1^ for lacCONS, pR and rrnBP1 promoters suggesting a relatively short-lived RPo is formed in case of the rrnBP1 (lifetime ~ 200 s), and a very long-lived, stable RPo is formed for pR promoter (lifetime ~ 2500 s) (Fig. 3B). This is consistent with previous measurements of open complex stability for rrnBP1 [37–39] and pR [40,41], further validating our assignment of the two components observed in this assay. Measurement of *k*_escape_ for the three promoters revealed values of ~ 0.014 s^−1^, 0.018 s^−1^, and 0.052 s^−1^ for lacCONS, pR, and rrnBP1, respectively (Fig. 3B). The relatively fast promoter escape kinetics observed for rrnBP1 compared to the other two promoters is expected as RPo stability has been demonstrated to be inversely correlated with promoter escape [25] and agrees well with previous biochemical experiments reporting a very early promoter escape event for the rrnBP1 promoter [42]. Similar experiments performed under conditions of crowding in CB revealed intensity vs time trajectories that fit to a biexponential with a fast (*k*_esc_; promoter escape) and a slow (*K*_dis_; RPo stability) component as before. Estimates of *k*_dis_ were similar for lacCONS and rrnBP1 (Fig. 3B) while that for pR was about 3-fold faster (~ 0.0014 s^−1^ in CB vs ~ 0.0004 s^−1^ in TB; Fig. 3B). Estimated rates of promoter escape in CB were ~ 0.020 s^−1^, 0.063 s^−1^, and 0.097 s^−1^ for lac-CONS, pR, and rrnBP1, respectively, indicating a modestly enhanced rate for lacCONS (~ 1.4-fold) and rrnBP1 (~ 1.2-fold), and a large enhancement of escape rate for pR (~ 5.4-fold) (Fig. 3B).

Like the analysis performed for estimating the viscosity-adjusted rates of unwound bubble formation, and assuming an inverse correlation of viscosity with rates of escape, we calculated viscosity-adjusted escape rates to gain further insight into the impact of excluded volume effect on promoter escape. The viscosity-adjusted escape rates in 1× TB were ~ 0.02 s^−1^, ~ 0.09 s^−1^, and ~ 0.03 s^−1^, and in 1× CB were ~ 0.16 s^−1^, ~ 0.49 s^−1^, and ~ 0.76 s^−1^, for lacCONS, rrnBP1 and pR promoters, respectively, indicating that the excluded volume effect produced by the crowded environment resulted in significant enhancement in escape kinetics for lacCONS (~ 6-fold), rrnBP1 (~ 5-fold), and pR (~ 24-fold) (Fig. 3C). Taken together our results suggest conditions of crowding have strikingly different extent of increase in the escape rates depending on the promoter sequence.

## Discussion

### Impact of excluded volume effect on promoter unwinding and promoter escape

The viscosity-adjusted rates obtained from our assays reflect the sole impact of the excluded volume effect produced by crowding. The viscosity-adjusted rates of unwound bubble formation under conditions of crowding reveal a modest increase for two promoters (~ 3-fold for lacCONS and rrnBP1) and similar values for pR (Fig. 2C). We note that the rate of unwound bubble formation in our assay reflects the rate of increase in unwound bubbles and as such depends both on the rate of the forward step of unwinding and the rate of the reverse step of rewinding – and that an increase in this rate may either result from an increase in the unwinding rate resulting from a lowered energy barrier for unwinding or a decrease in the rewinding rate resulting from a raised energy barrier for rewinding. Interestingly, the RPo maturation rates were not significantly altered suggesting the conformational changes involved in these late stages of RPo formation are not impacted in presence of crowders.

On the other hand, the viscosity-adjusted rates of promoter escape showed a very large enhancement under conditions of crowding with ~ 6-fold, ~ 5-fold, and ~ 24-fold increase for lacCONS, rrnBP1 and pR promoters (Fig. 3C). Notably we observe a significant decrease in RNAP-promoter stability for all three promoters with viscosity-adjusted dissociation rates increasing by ~ 4.6-fold, ~ 4.3-fold and ~ 14.4-fold for lacCONS, rrnBP1 and pR, respectively. Therefore, the observed impact of the excluded volume effect on all three promoters is consistent with the idea that the stability of a RNAP-promoter open complex (RPo) is inversely correlated with promoter escape rates since a weaker RPo would result in a lower energy barrier to disruption of RNAP-promoter contacts [25].

The effect of crowders on viscosity-adjusted rates of promoter unwinding and promoter escape differ significantly for the three different promoters. It is tempting to correlate these observations relative to difference between promoter sequence elements like the -10/-35 hexamers, the spacer, the discriminator sequence and/or the initial transcribed sequence. However, we note that the overall rates of full promoter unwinding as measured here depend on all steps that lead to unwinding and include recognition of the -10 hexamer, initial nucleation of unwinding involving flipping out of the conserved -11A in the non-template strand, full unwinding, and the rate of rewinding. Similarly, the rates of promoter escape as measured here depend on all steps after unwound bubble formation including initiation of RNA synthesis, abortive initiation, initiation pausing, late off-pathway events in initial transcription, and finally breaking of RNAP-promoter contacts resulting in formation of mature elongation complexes. The rates of each of these individual substeps can vary widely for different promoter sequences and given the large variations observed here it is not possible to identify the exact impact of crowding on these individual sub-steps. We propose the effect of promoter sequence determinants on crowding needs to be investigated using large libraries of promoters and high throughput assays connecting function to sequence as described in [43].

### Overall effect of crowding on transcription initiation

We note that the overall kinetics of processes captured in our assays are affected by the presence of crowders in two ways: (a) an increase in viscosity, and (b) the excluded volume effect. Increased viscosity typically leads to a slowdown in the kinetics [4,32]. Here, we find that excluded volume effect has an opposite effect as it results in enhanced rates of both unwound bubble formation and promoter escape, albeit to different degrees. Since the observed increase is modest for unwound bubble formation the slowdown of kinetics due to increased viscosity dominates resulting in an overall decrease in the rate of unwound bubble formation (Fig. 2B). In contrast, the observed increase in the rates of promoter escape due to excluded volume effect are large and therefore becomes the dominating factor. Therefore, the measured overall rates of promoter escape are faster for all three promoters (Fig. 3B).

**Fig. 4:**
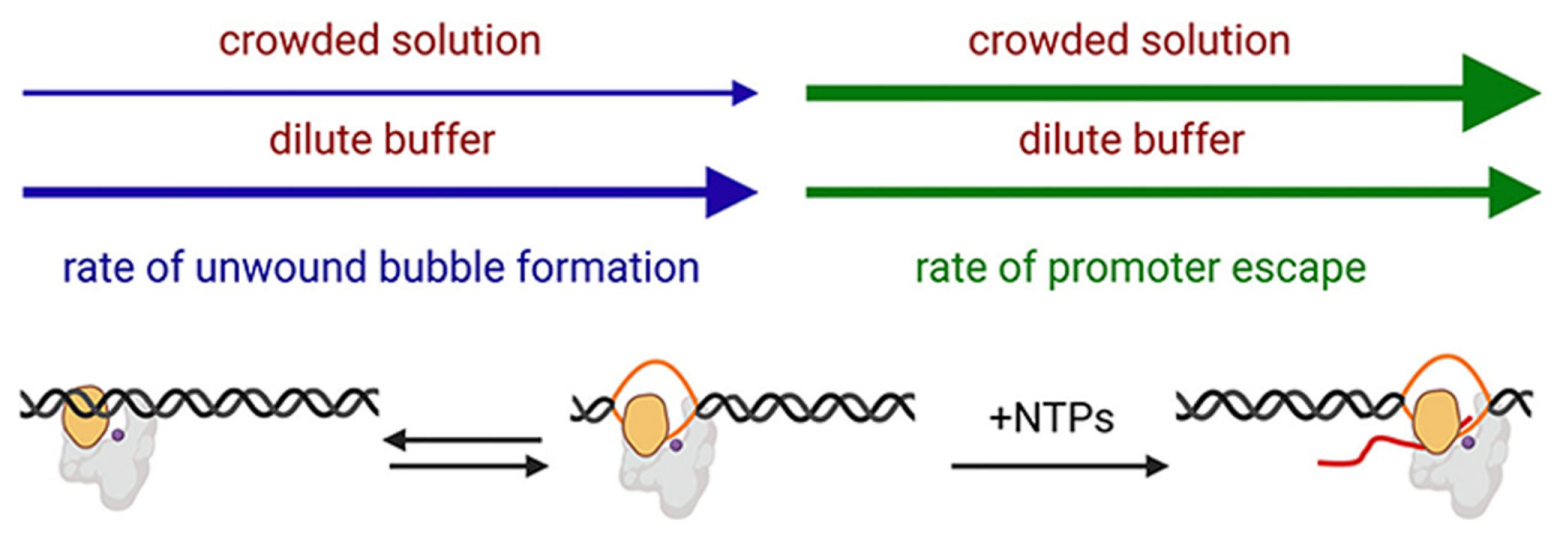
Crowded environments affect the kinetics of unwound bubble formation and promoter escape in opposing ways. Grey, RNAP; orange, RNAP clamp; purple dot, RNAP active-centre; black, ds-DNA; orange, ss-DNA; red, RNA; blue arrow, rate of unwound bubble formation; green arrow, rate of promoter escape.

We argue that although it is important to understand the impact produced due to excluded volume effects alone, it is the overall impact of crowded solutions that is important to understand and that it is the overall rates, which combine both the effects produced by enhanced viscosity of the medium and excluded volume effect that is important for commenting on the effect of crowded conditions on transcription initiation kinetics. Therefore, our findings suggest that crowding has opposing effects on two crucial steps in RNA synthesis: it decreases the rate of unwound bubble formation while increasing the rate of promoter escape (Fig. 4). Importantly, both effects are highly promoter-specific. We suggest for promoters where unwound bubble formation is rate-limiting, crowding would lead to an overall decrease in RNA synthesis, and conversely, for promoters where escape is rate-limiting, crowding would cause an overall increase in RNA synthesis. In cases where both unwinding and escape rates are comparable, kinetic models incorporating the individual effects on each step are needed to predict the overall impact of crowding on RNA synthesis [44]. Such a promoter-dependent impact on transcription suggests altered conditions of crowding inside a cell can result in global changes. Consistent with this idea it has been reported that the extent of macromolecular crowding inside an *E. coli* can change under osmotic stress [45] – a condition which results in upregulation or downregulation of ~ 300 genes in *E. coli* [46–48].

It is important to remember that our observations are based on effects produced by a single crowding agent of moderate size (~ 2.45 nm; [28]) compared to the size of the RNAP-promoter complex, and since variations in size of the crowder likely influence the extent of excluded volume effect, it is important to investigate in a future study kinetics of sub-steps in transcription initiation in presence of a variety of crowding agents of different size and nature to generate further insights.

## Supplementary Material

SI

## Data Availability

The data that support the findings of this study are included in this manuscript. Any additional data are available from the corresponding author [abhishek@iicb.res.in] upon reasonable request.

## Abbreviations

CB: crowding buffer
FCS: fluorescence correlation spectroscopy
PEG: polyethylene glycol
PIFE: photoisomerisation-induced fluorescence enhancement
RNAP: RNA polymerase
RPo: RNAP-promoter open complex
TB: transcription buffer
