# Supplementary material for: Macromolecular crowding has opposite effects on two critical sub-steps of transcription initiation
Source: FEBS Lett. Author manuscript; Available in PMC 2024 May 10. (PMC7615953; doi:10.1002/1873-3468.14851)
Supplement: SI [file EMS195117-supplement-SI.docx]

**SUPPLEMENTARY INFORMATION**

**Macromolecular crowding results in opposite effects on two critical sub-steps of transcription initiation**

Pratip Mukherjee^a^, and Abhishek Mazumder^a^*

^a^Structural Biology and Bioinformatics Division, CSIR-Indian Institute of Chemical Biology, 4 Raja S. C. Mullick Road, Jadavpur, Kolkata-700032

*To whom correspondence should be addressed: [abhishek@iicb.res.in](mailto:abhishek@iicb.res.in)

**Table S1. Sequence of Cy3 labelled promoter constructs.**

Non-template strands are at top and template strands are bottom; promoter sequence elements -35 region and -10 region are in blue, Cy3 labelled nucleotide is in green, and promoter bubble segments are shown inside a dashed box.


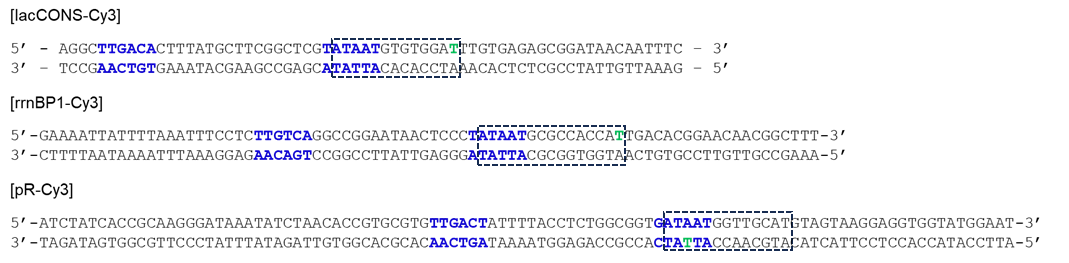


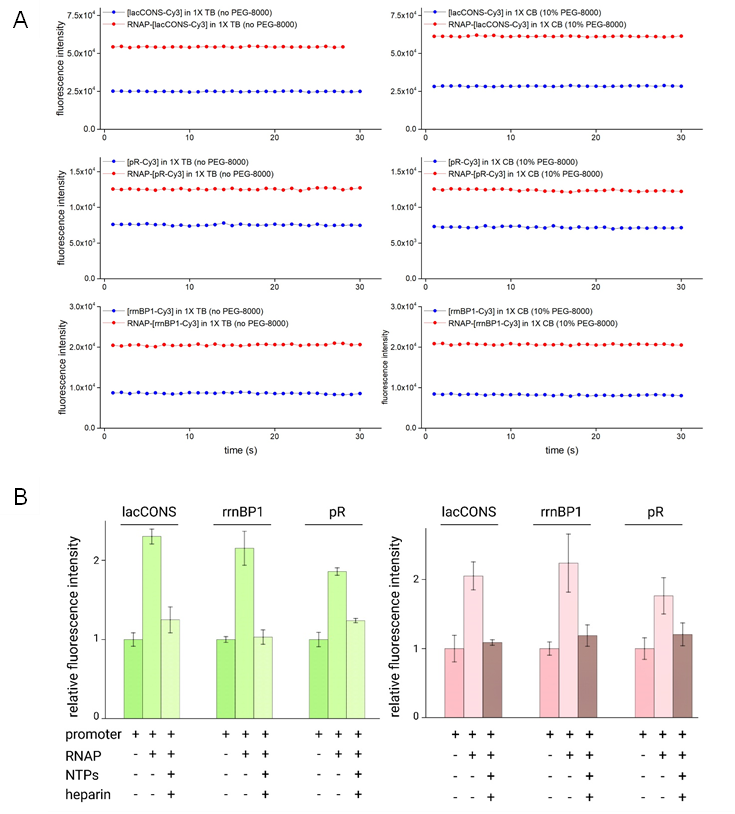


**Figure S1. Results from endpoint PIFE experiments. (A)** Representative measurements recorded for the endpoint assay showing the fluorescence intensity change after RNAP binds to DNA in absence and presence of 10% PEG-8000. Data for promoter DNA only are in blue and for RNAP-promoter complexes are in red. (B) Bar plot showing relative fluorescence intensity from [lacCONS-Cy3], [rrnBP1-Cy3], and [pR-Cy3] promoter fragments, deep green (1X TB) or magenta (1X CB) bars; RPo formed with [lacCONS-Cy3], [rrnBP1-Cy3], and [pR-Cy3] promoter fragments with RNAP holoenzyme for 40 mins at 37°C in 1X TB or 1X CB, green(1X TB) or light magenta (1X CB) bars; elongation complex (RDe) formed after incubation of the respective RPo fragments with 200 μM each ATP, CTP, GTP, UTP and 50 μg/ml heparin for 40 mins, light green (1X TB) or deep magenta (1X CB) bars. Experiments were repeated three times, error bars are showing standard deviations.

**
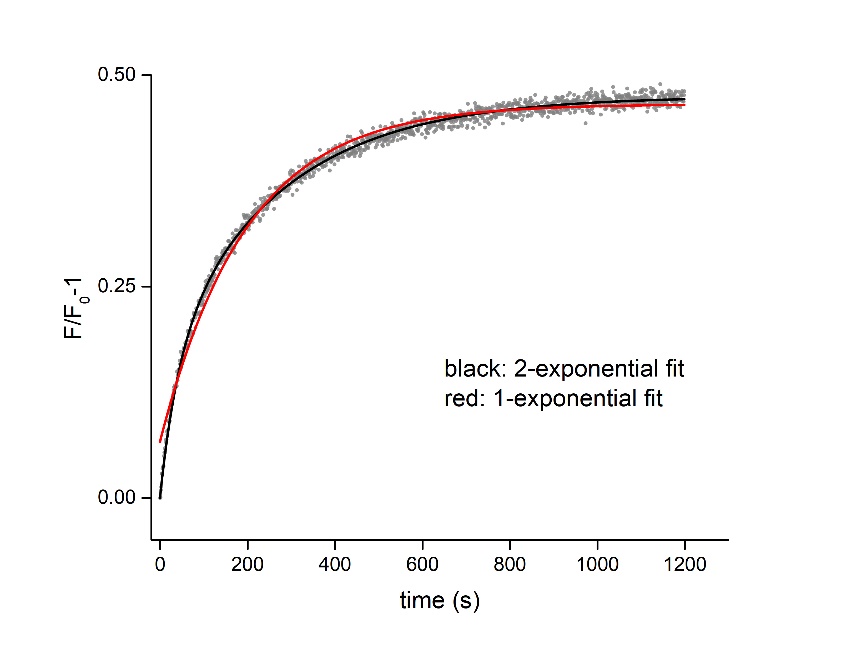

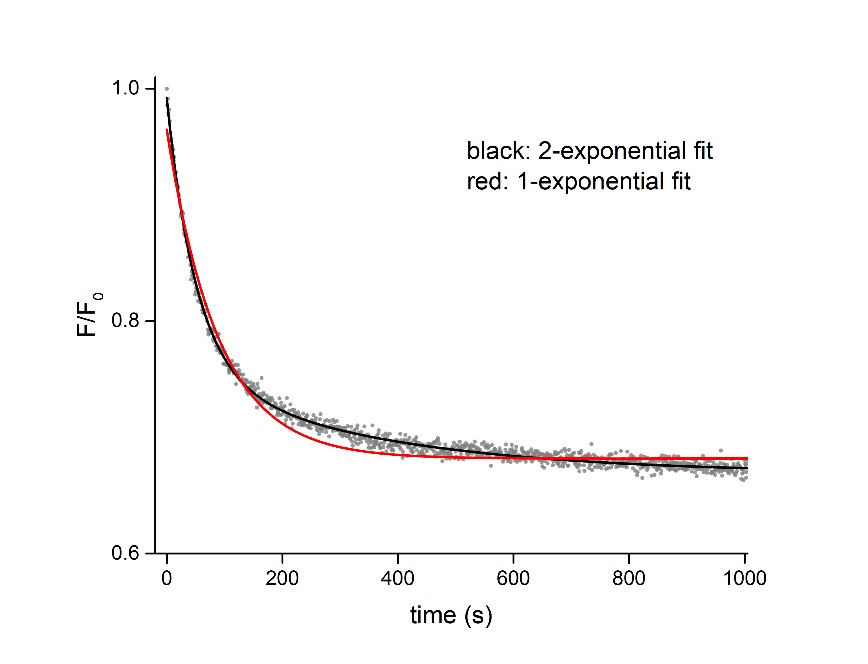
Figure S2:** Representative example showing a 1-exponential (red) and a 2-exponential (black) fit to promoter unwinding (left) and promoter escape (right) experiment using the lacCONS promoter fragment.


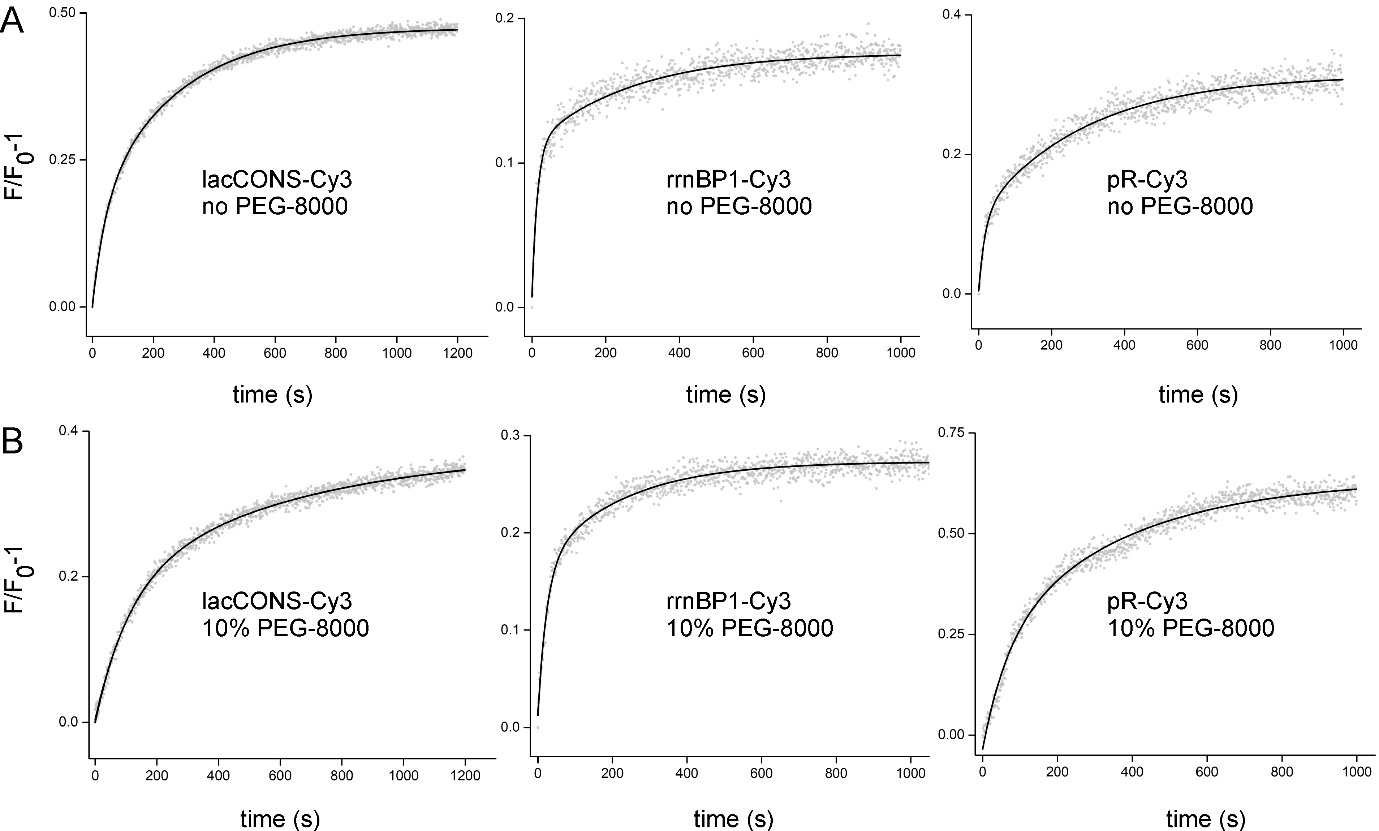


**Figure S3.** Full time-trajectories recorded for promoter unwinding PIFE experiments. (**A**) Experiments performed in 1X transcription buffer with lacCONS-Cy3, rrnBP1-Cy3 and pR-Cy3 promoter fragments. (**B**) Experiments performed in 1X crowding buffer containing 10% PEG-8000 with lacCONS-Cy3, rrnBP1-Cy3 and pR-Cy3 promoter fragments. Gray: raw data points; Black line: 2-exponential fit to the data. Excitation was at 550 nm and emission was recorded at 570 nm. Slit widths were 5 nm and integration time was 1 second. Experiments were repeated three times.


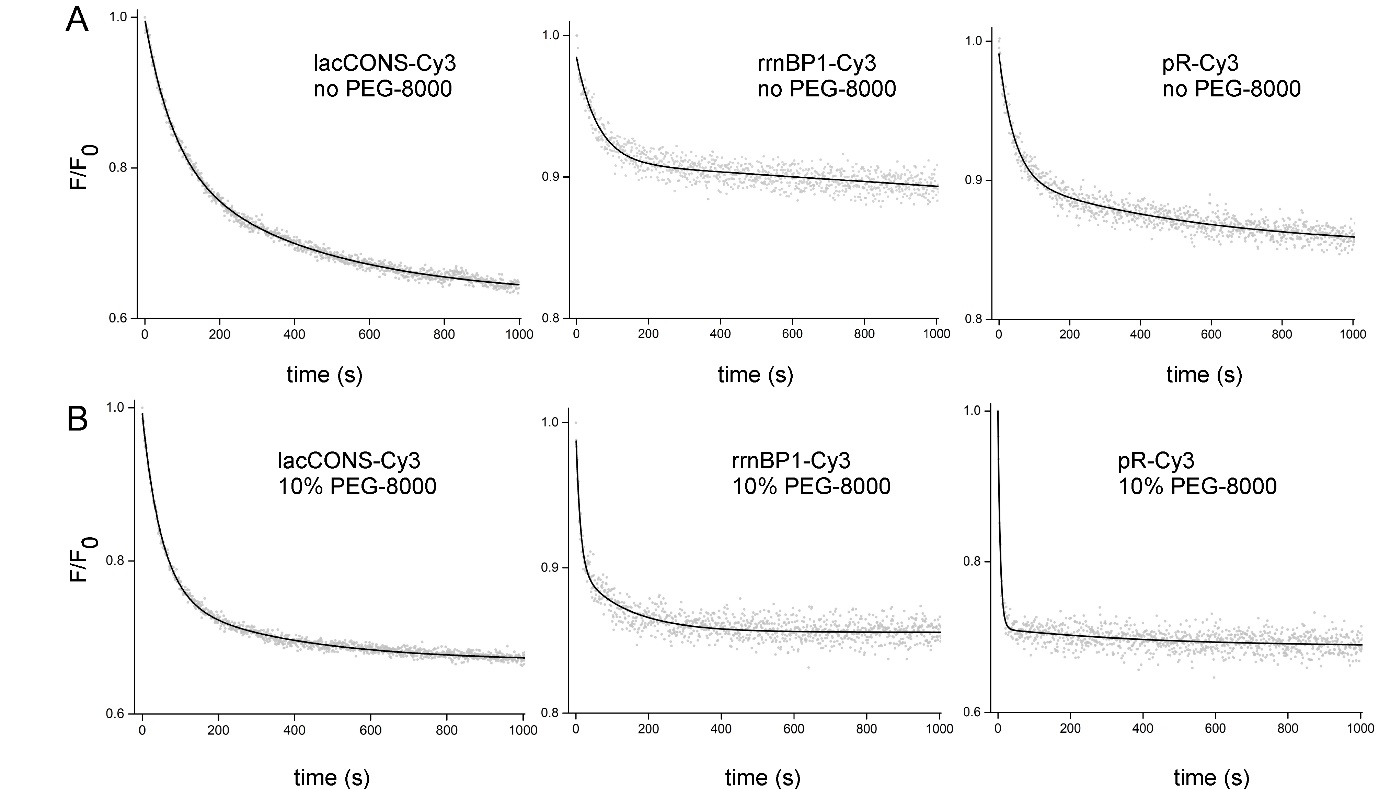


**Figure S4.** Full time-trajectories recorded for promoter escape PIFE experiments. (**A**) Experiments performed in 1X transcription buffer with lacCONS-Cy3, rrnBP1-Cy3 and pR-Cy3 promoter fragments. (**B**) Experiments performed in 1X crowding buffer containing 10% PEG-8000 with lacCONS-Cy3, rrnBP1-Cy3 and pR-Cy3 promoter fragments. Gray: raw data points; Black line: 2-exponential fit to the data. Excitation was at 550 nm and emission was recorded at 570 nm. Slit widths were 5 nm and integration time was 1 second. Experiments were repeated three times.

**
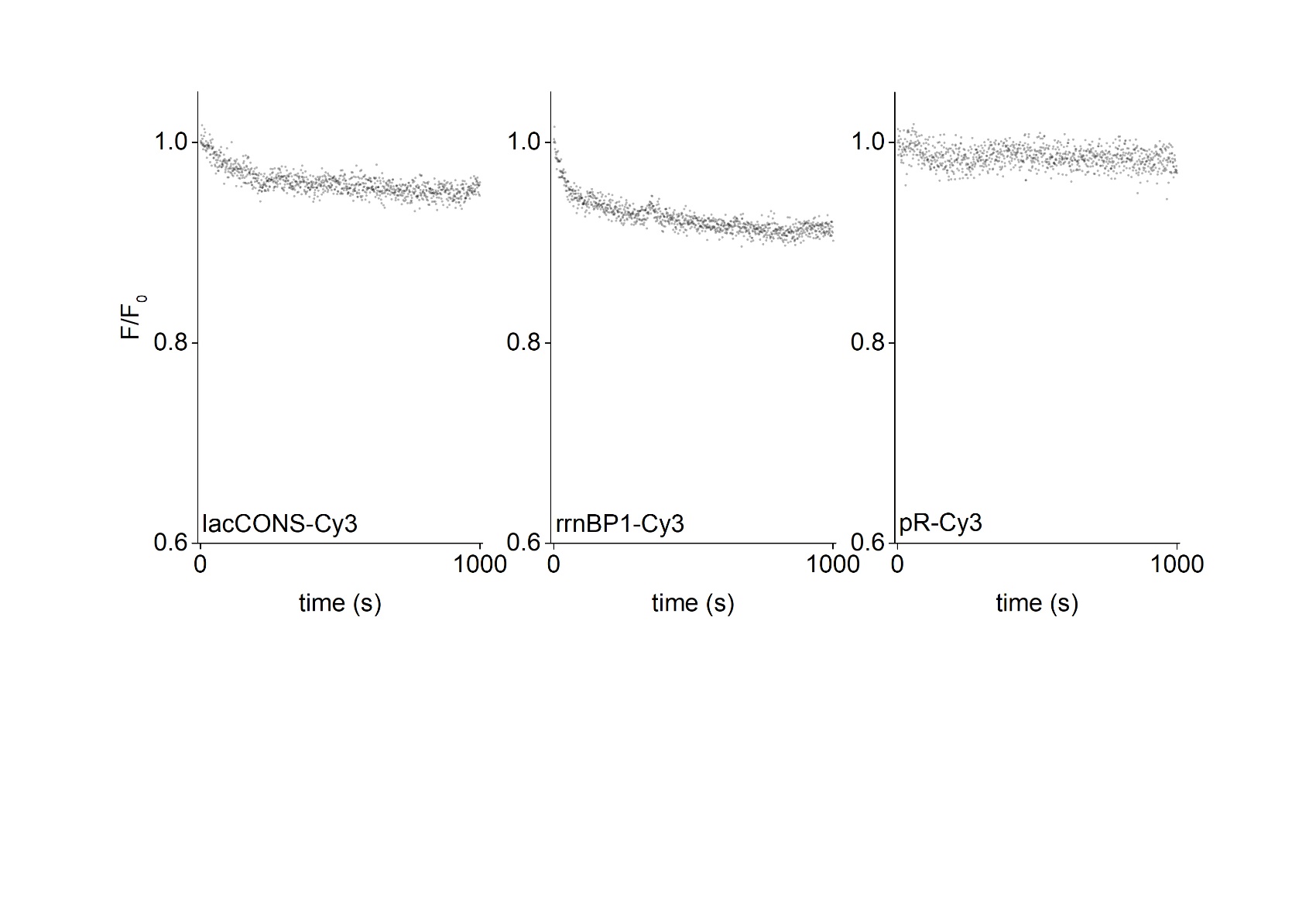
**

**Figure S5.** Representative time course of relative fluorescence intensity changes from Cy3 attached to the promoter bubble of a consensus bacterial promoter, lacCONS (*left*), rrnBP1 (*middle*), pR (*right*) obtained after manual mixing of RNAP-promoter open complex (RPo) with 50 μg/ml heparin in 1X transcription buffer (TB; grey dots). Time resolution: 1 second; excitation: 550 nm; emission: 570 nm.
